# Surface states in bulk single crystal of topological semimetal Co_3_Sn_2_S_2_ toward water oxidation

**DOI:** 10.1126/sciadv.aaw9867

**Published:** 2019-08-16

**Authors:** Guowei Li, Qiunan Xu, Wujun Shi, Chenguang Fu, Lin Jiao, Machteld E. Kamminga, Mingquan Yu, Harun Tüysüz, Nitesh Kumar, Vicky Süß, Rana Saha, Abhay K. Srivastava, Steffen Wirth, Gudrun Auffermann, Johannes Gooth, Stuart Parkin, Yan Sun, Enke Liu, Claudia Felser

**Affiliations:** 1 Max Planck Institute for Chemical Physics of Solids, 01187 Dresden, Germany.; 2 School of Physical Science and Technology, ShanghaiTech University, 201203 Shanghai, China.; 3 Zernike Institute for Advanced Materials, University of Groningen, 9747 AG, Groningen, Netherlands.; 4 Max Planck Institute for Coal Research, Kaiser-Wilhelm-Platz 1, 45470 Mülheim an der Ruhr, Germany.; 5 Max Planck Institute for Microstructure Physics, 06120 Halle, Germany.; 6 Institute of Physics, Chinese Academy of Sciences, 100190 Beijing, China.

## Abstract

The band inversion in topological phase matters bring exotic physical properties such as the topologically protected surface states (TSS). They strongly influence the surface electronic structures of the materials and could serve as a good platform to gain insight into the surface reactions. Here we synthesized high-quality bulk single crystals of Co_3_Sn_2_S_2_ that naturally hosts the band structure of a topological semimetal. This guarantees the existence of robust TSS from the Co atoms. Co_3_Sn_2_S_2_ crystals expose their Kagome lattice that constructed by Co atoms and have high electrical conductivity. They serves as catalytic centers for oxygen evolution process (OER), making bonding and electron transfer more efficient due to the partially filled orbital. The bulk single crystal exhibits outstanding OER catalytic performance, although the surface area is much smaller than that of Co-based nanostructured catalysts. Our findings emphasize the importance of tailoring TSS for the rational design of high-activity electrocatalysts.

## Teaser

Co_3_Sn_2_S_2_ hosts Co atom–derived surface states at the Kagome lattice, favoring the electron transfer process in oxygen evolution.

## INTRODUCTION

Heterogeneous catalytic reactions such as electrochemical water spitting are closely related to the surface electronic structures of the catalysts, such as the surface states and surface atomic termination (*1*, *2*). The topological phase materials, with rich exotic physical properties, provide an ideal platform to explore the interplay between surface states, electron transfer, and surface catalytic reactions (*3*, *4*). Three-dimensional (3D) topological insulators have robust metallic surface states that cover the entire material. Unlike the easily destroyable surface states derived from dangling bonds, vacancies, or doping, topological surface states (TSSs) are a result of the inversion of the bulk bands at the surface. Thus, they are robust against surface modifications and defects (*5*–*7*). The electron spin is in a lock-up state with its momentum due to the spin-orbit coupling at the crystal surface. This could notably depress backscattering and Anderson localization of conduction electrons, which are imperative for materials that are always accompanied with, to some extent, surface defects (*8*). However, the insulating properties of topological insulators are a challenge for the electron migration when used as electrocatalysts, which will lead to lower apparent catalytic activity in comparison with that of a highly conducting catalyst (*9*). Topological semimetals such as Weyl semimetals and topological nodal line semimetals are good candidates for electrocatalysis due to their high conductivity, nontrivial topologically protected surface states, and suitable carrier density around Fermi level (*7*).

The oxygen evolution process (OER) is a kinetically sluggish process that involves the formation of bonds and electron transfer between the catalytic sites and the adsorbates (*10*, *11*). Thus, the reaction kinetics are jointly controlled by the geometric properties (size, shape, crystallinity, etc.) and electronic structure (work functions, *d*-band center positions, spins, etc.) of the catalysts. Recently, *e*_g_ orbital filling and spin states of the OER active sites have been identified to be a reasonable descriptor of catalytic activity based on the idea that the *e*_g_ orbitals can form strong bonds with the oxygenated adsorbates (*12*). It is found that depending on the spin states of the transferred electrons, either ground-state triplet oxygen molecule or hydrogen peroxide can be produced. This greatly affects the needed overpotential to drive the reaction (*13*). Thus, it is expected that excellent OER catalytic activities can be achieved by introducing elemental vacancies, applying strain, or tuning transition-metal coordination and spin states (*14*–*17*). However, the strategies based on extrinsic modifications are inevitably accompanied with crystal collapse and distortion, making the exploration of their influence on catalytic activity more difficult. Here, taking the bulk single crystal of the topological semimetal Co_3_Sn_2_S_2_ as a proof-of-concept study, we demonstrate a unique strategy to combine the advantages of a normal semimetal and a topological insulator with robust surface states, which could notably enhance the OER kinetics. Co_3_Sn_2_S_2_ was recently discovered as the first magnetic Weyl semimetal with time-reversal symmetry breaking, showing a giant anomalous Hall effect in the bulk and potential TSSs on the crystalline surface (*18*, *19*). At room temperature, the band structure of Co_3_Sn_2_S_2_ naturally hosts the electronic structure of a topological semimetal. We observed high conductivity, as well as robust surface states, derived by Co atoms on the Kagome lattices and located just above the Fermi level. The *e*_g_ orbital of the surface Co atoms is partially filled and points to the *p* orbital of the adsorbed hydroxide ions, thus favoring electron transfer and strengthening the bonds between the adsorbate and catalytic sites. When used as an electrocatalyst for the OER, the bulk single-crystal Co_3_Sn_2_S_2_ shows high activity and comparable to that of reported Co-based nanostructures with a much larger surface area. The present work reveals a valuable method to develop an efficient OER electrocatalyst by manipulating the surface states and spin states.

## RESULTS

### Motivation

**Fig. 1:**
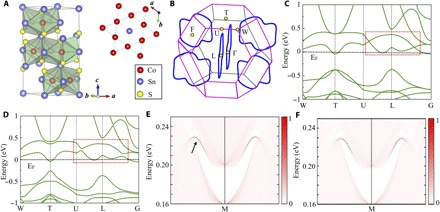
Crystal and band structure of bulk single-crystal Co_3_Sn_2_S_2_. (**A**) Crystal structure of Co_3_Sn_2_S_2_ obtained from single-crystal XRD and the Kagome lattice structure constructed by Co atoms in the *a*-*b* plane. (**B**) The 3D BZ projected in the (001) direction. Three pairs of nodal lines are shown in the first BZ. (**C**) Band structure of Co_3_Sn_2_S_2_ in a paramagnetic state without the consideration of SOC effect. The band linear crossing near the Fermi energy can be found around the point L. (**D**) Band structure of Co_3_Sn_2_S_2_ with the inclusion of SOC effect. The band linear crossing is open, resulting in the band gap. (**E**) The nontrivial surface states on (001) facet of Co_3_Sn_2_S_2_ crystal with Sn termination, which is not fully occupied and located just above the Fermi level. (**F**) The contribution of Co atoms to the nontrivial surface states shown in (E). Nearly all the states originate from the surface Co Kagome layer.

Co_3_Sn_2_S_2_ is chosen in this study because of the following reasons: (i) Co_3_Sn_2_S_2_ is the first experimentally confirmed magnetic Weyl semimetal, with the existence of a Co atom–derived topologically protected surface states (*18*). (ii) Co atoms generally serve as active centers for the OER (*20*–*22*). (iii) The observed high conductivity, robust surface states, and the magnetic Co ions in this compound indicate an intrinsic high OER activity (*19*, *23*). The crystal structure of Co_3_Sn_2_S_2_ is shown in Fig. 1A. It belongs to the Shandite family, exhibiting hexagonal Kagome lattices with the space group 166 (R-3*m*). Co atoms occupy Wyckoff position 3e, while S atoms are located in position 2c with *z*(S) = 0.216. There are two types of Sn atoms, which occupy positions 1a and 1b (*23*). This arrangement can be viewed as a quasi-2D structure stacked along the *z* direction with the Sn-[S-(Co_3_Sn)-S] layer group. The Co atoms form a Kagome lattice network with one Sn atom located at the center (Fig. 1A, top right). Thus, freshly cleaved surfaces of the measured single crystals for catalysis always expose the (001) facet, with three different terminations: (i) six Sn atoms, (ii) six S atoms, and (iii) the Kagome lattice with six Co atoms and one Sn atom. The 3D Brillouin zone (BZ) and the corresponding (001) surface BZ are shown in Fig. 1B. The calculation details can be seen in the Supplementary Materials.

To predict the room-temperature electrochemical behavior, we analyzed the electronic structure of Co_3_Sn_2_S_2_ in the paramagnetic state. From the ionic picture, because of the nonclosed shell configuration of the valence electrons (3 × Co − 3*d*^7^ + 2 × Sn − 5*p*^2^ + 2 × S − 3*p*^4^), a metallic nature is expected in this compound. The charge-carrier density is determined to be around 1.22 × 10^21^ cm^−3^, showing the semimetallic characteristics. We show that the band structure in the paramagnetic state without spin-orbital coupling (SOC), as depicted in Fig. 1C, shows the electronic band structure without inclusion of SOC. Because of the crystal mirror symmetry, the band inversion induced the linear crossing near the Fermi energy can be found around the L point of the BZ, which is consistent with the reported Weyl semimetal state in ferromagnetic Co_3_Sn_2_S_2_ (*19*). These nodal lines are gapped everywhere by SOC, as shown in Fig. 1D and fig. S1, allowing to define a Z_2_ invariances. As given in Table 1, a nonzero Z_2_ of (1; 000) can be found in Co_3_Sn_2_S_2_ if the Fermi level is located within the band gap exactly. Subsequently, we investigated the surface band structure with Sn and S terminations. Figure 1E shows the nontrivial surface states on the (001) facet for Sn termination, as calculated by using Green’s function based on the tight-binding Hamiltonian. Nontrivial surface states can be observed but are difficult to distinguish because of the weak SOC effect. With increasing strength of the SOC, the upper surface states become nontrivial here (fig. S2, surface states with 2SOC and 3SOC). Furthermore, we calculated the contribution of the Co (Fig. 1F), Sn, and S atoms (fig. S3) to the nontrivial surface states (surface states with S and Sn) and found that almost all of the surface states are derived from the Co atoms. The same results were obtained when exposing the S layer (fig. S4). Further orbital analysis indicated that almost all of these surface states are almost from the Co *d* orbital. These TSSs are unoccupied and located only 0.23 eV above the Fermi level, in addition to being nontrivial and robustness against static perturbations that preserve the relevant symmetries. Last, we calculated the decay depth of the surface states, which is estimated to be approximately 30 unit cells in the bulk. All these observations suggest that Co_3_Sn_2_S_2_ is the ideal candidate to be used for exploring the electron transfer kinetics in the water oxidation process.

**Table 1:** Z_2_ numbers (1; 000) of Co_3_Sn_2_S_2_ crystal. The product of parity of occupied bands at each time reversal invariant momenta (TRIM) points.

**TRIM points**	**𝚪 (0,0,0)**	**L (0.5,0,0) × 3**	**F (0.5,0.5,0) × 3**	**T (0.5,0.5,0.5)**
Parity	−	−	+	−

### Electrochemical behavior on bulk single-crystal surface

**Fig. 2:**
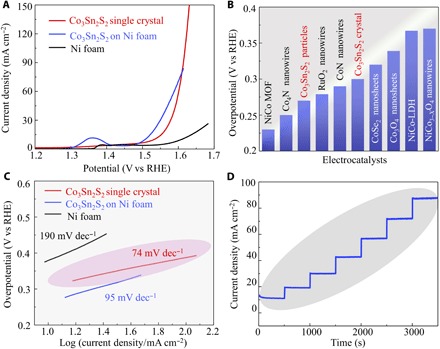
Electrochemical performance of Co_3_Sn_2_S_2_ single-crystal catalyst. (**A**) OER polarization curves for Ni foam, Co_3_Sn_2_S_2_ single crystal, and Co_3_Sn_2_S_2_ micropowder crushed from the single crystal. (**B**) Overpotential of Co_3_Sn_2_S_2_ single-crystal catalyst at 10 mA cm^−2^ compared with some recently reported results for OER electrocatalysts. (**C**) Tafel plot of Ni foam, Co_3_Sn_2_S_2_ single crystal and Co_3_Sn_2_S_2_ micropowder Koutecky-Levich plots in O_2_-saturated 1 M KOH solution. The wide linear regime indicates the excellent electron transfer kinetics even at large overpotential. RHE, reverse hydrogen electrode. (**D**) Multicurrent process with the current density increased from 10 to 85 mA cm^−2^ without iR correction.

To confirm the role of *d*-derived surface states from Co atoms, high-quality bulk single crystals with the desired surface terminations are required. Here, we developed a self-flux method for the synthesis of large-size Co_3_Sn_2_S_2_ single crystals (*18*). OER activities were measured in a conventional three-electrode cell containing 1 M KOH solution at a low scan rate of 5 mV s^−1^ to minimize capacitive currents. A cuboid-shaped bulk single crystal was attached to a Cu wire by silver paint and used as the working electrode. Figure 2A shows the *i*R-corrected linear sweep voltammogram (LSV) curve of the bulk single crystal. When the thermodynamic OER potential (*E*^0^ H_2_O/O_2_ = 1.23 V) is used as the reference, an overpotential of just 300 mV is required to reach a current density of 10 mA cm^−2^. This value is close to, or even smaller than that for, nanostructured electrocatalysts with a considerably larger surface area (Fig. 2B), such as (Ni/Co)_0.85_Se nanotube arrays (255 mV) (*24*), CoN nanowires (290 mV) (*25*),CoSn_2_ nanocrystals (299 mV) (*26*), and NiCo metal-organic framework nanosheets (371 mV) (*27*–*29*). Crushing the bulk single crystal into small particles and deposited these onto Ni foam results in a further enhanced OER performance with an overpotential of 270 mV at 10 mA cm^−2^. The poor activity of Ni foam and Cu wire with silver paint suggests that the high catalytic activity of this sample originates from the Co_3_Sn_2_S_2_ phase (fig. S5).

The catalytic kinetics for oxygen evolution is assessed by analysis of the corresponding Tafel plots. As shown in Fig. 2C, the resultant Tafel slope of the Co_3_Sn_2_S_2_ single crystal is only 74 mV dec^−1^, which is significantly lower than that of Ni foam (190 mV dec^−1^) and Co_3_Sn_2_S_2_ microcrystals (95 mV dec^−1^), despite the fact that the latter two catalysts have much smaller surface areas. This result indicates the superior reaction kinetics on the bulk single-crystal catalyst. In addition, it is interesting to observe that the linear region of the Tafel slope is much wider than for most reported studies. It is well established that the Tafel analysis is based on the Butler-Volmer equation under the assumption of constant coverage of the intermediate species. However, both surface coverage of the intermediate species and the reaction constant are strongly potential dependent (*30*). This well explains the rapid increase of the Tafel slope in the high-overpotential range. For Pt in 1 M KOH, this value increases from 60 to 120 mV dec^−1^ when increasing the applied potential (*31*, *32*). The validity of the Butler-Volmer equation at such a large applied potential suggests a fast electron-transfer kinetics on the bulk single-crystal surface. We performed multistep chronopotentiometry measurements to characterize the kinetic behavior of OH group insertion. As shown in Fig. 2D, the current densities show a rapid response to the applied potential and remain stable in the following 500-s test. This suggests a fast charge-transfer and mass transport process, with the active species (OH^−^) at the crystal surface are oxidized rapidly when the potential is abruptly changed (*33*). The 12-hour durability test reveals the high stability of the microcrystal with negligible loss of the anodic current (fig. S6A). This is further confirmed by the imperceptible variation in the LSV curve after the stability test (fig. S6B).

### Phase and physical properties

**Fig. 3:**
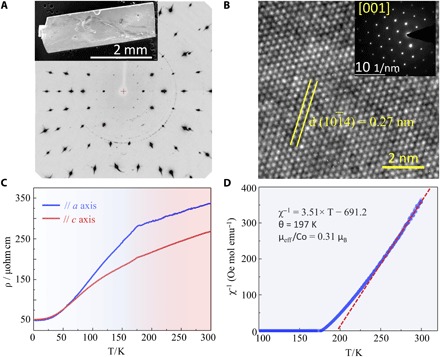
Phase structure and physical properties of Co_3_Sn_2_S_2_ single-crystal catalyst. (**A**) Single-crystal XRD pattern of Co_3_Sn_2_S_2_. The pattern was recorded by rocking by 32° about the *b* axis of the rhombohedral cell. The high quality of the crystal is proven by the clear and sharp diffraction spots. The faint rings may be attributed to distortions and contaminations on the crystal surface. A typical SEM image of the single crystal is shown in the upper left corner. (**B**) HRTEM image of the Co_3_Sn_2_S_2_ single crystal prepared using the FIB technique and the SAED pattern recorded along the [001] crystal orientation. (**C**) Temperature dependence of electric resistivity of Co_3_Sn_2_S_2_ single crystal in zero field. The current was applied along the *a* and *c* axes. (**D**) Reciprocal susceptibility as a function of temperature. The magnetic moments are derived from Co atoms in the Kagome lattice. Using Curie Weiss law, an effective Bohr magneton μ_eff_ of 0.31 μ_B_/Co is obtained.

To understand the excellent OER activity in Shandite Co_3_Sn_2_S_2_, the phase and physical properties of the bulk single crystal are investigated in detail. Figure 3A and fig. S7 show the scanning electron microscope (SEM) image of a typical crystal for physical and electrocatalysis measurements. Bulk single crystals with dimensions of up to several centimeters can be grown and can easily be exfoliated into lamella. The sharp and clear ordered diffraction spots from the Laue diffraction pattern confirmed the high quality of the single crystal (Fig. 3A). This high quality and the purity were further confirmed by powder x-ray diffraction (XRD) and energy-dispersive x-ray spectroscopy (EDS) spectra (figs. S8 and S9). A possible structural transition is excluded by performing single-crystal XRD measurements down to 100 K using a Bruker D8 Venture diffractometer. As revealed by the refinement parameters (table S1), the R-3*m* space group is maintained throughout the measured temperature range, with only a slight thermal expansion. As shown in fig. S10 (data S1 and S2), the crystal structure at 100 K is characteristic to the Shandite family; the Co atoms, which are octahedrally coordinated by four Sn and two S atoms, form a Kagome net perpendicular to the *c* axis. However, the cobalt-centered octahedra are compressed, with significantly shorter Co-S distances (2.17 Å) than the Co-Sn (2.67 Å) distances (table S3). For high-resolution transmission electron microscopy (HRTEM) observations, a thin lamella was fabricated by focused ion beam (FIB) micromachining. As shown in Fig. 3B and fig. S11, the well-defined lattice fringes with a spacing of 0.27 nm could be readily indexed to the ($10\bar{1}4$) plane of hexagonal Co_3_Sn_2_S_2_. The exposed surface is (001), as confirmed by the selected-area electron diffraction (SAED) pattern that was recorded along the [001] direction (inset in Fig. 3B). The temperature (*T*) dependence of resistivity measurements was measured in four-probe configuration from 2 to 300 K along *a* and *c* axes, respectively. The low anisotropy of the electrical resistivity indicated that the Co *d* electrons are itinerant. The room-temperature resistivity along the *a* axis [exposing the (001) surface] was only 337 μohm cm, which is much lower than that for nanostructured electrocatalysts deposited on an electrode or conductive substrates (*16*, *34*). The carrier concentration is determined to be 1.22 × 10^21^ cm^−3^ by Hall measurement. The combination of high conductivity and high carrier concentration in the single crystal could improve the electrocatalytic activity remarkably (*35*). The Curie temperature (*T*_C_) was determined to be 175 K from zero-field cooling (ZFC) and field cooling (FC) measurements of magnetization (fig. S12). Susceptibility follows the Curie-Weiss law, and an effective moment of 0.31 μ_B_/Co was observed (Fig. 3D). This is consistent with the results of band structure calculations and the fact that the band dispersion near *E*_F_ is mainly dominated by the Co 3*d* orbitals and having a polarized magnetic moment of about 0.33 μ_B_/Co.

### Surface electronic structures

**Fig. 4:**
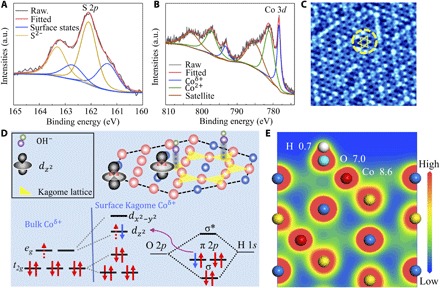
Surface structure and OER mechanism. (**A**) Detailed XPS analysis of the prepared single crystal. High-resolution XPS spectra for (A) S 2*p* and (**B**) Co 3*d*. (**C**) STM topography of a cleaved Co_3_Sn_2_S_2_ single-crystal thin flake showing an area of 8 nm by 8 nm. The Kagome lattice is highlighted by yellow lines in the circle. (**D**) Schematic representation of the favored OH uptake with the Co 3*d* orbitals. The exfoliation between the S-Sn plane break the octahedral symmetry of the surface Co atoms in the Kagome lattice (highlighted by yellow triangle). The empty 3*d*_z^2^_ orbital points to the *p* orbital of the OH group, resulting in a strong bonding between them. (**E**) Contour plots of the total charge distribution of Co_3_Sn_2_S_2_ single crystal with one OH group bonded to the Co atoms. Electronic charges are distributed in the vicinity of Sn atoms. However, for Co atoms, one can see the electron transfer through the Co─O bonding. a.u., arbitrary units.

High-resolution x-ray photoelectron spectroscopy (XPS) provides additional surface information for single-crystal catalysts (fig. S13). As shown in Fig. 4A, the S *p*_3/2_ band with a binding energy (BE) of 162.1 eV corresponds to the S^2−^ configuration in Co_3_Sn_2_S_2_. This value is lower than that of its counterpart Ni_3_Sn_2_S_2_ (162.8 eV), which has been confirmed to adopt the electronic configuration (Ni^0^)_3_(Sn^2+^)_2_(S^2−^)_2_, suggesting a partial positive charge on Co atoms (*36*). In addition, a shoulder peak with a BE of 161.4 eV is observed and can be interpreted as a surface-derived contribution (*37*). This indicates the exposure of the S_2_-Co-Sn_4_ octahedra when exfoliating the bulk crystal. High-resolution Co 3*d* spectra provide more interesting information, as shown in Fig. 4B. The peak at BE = 778.3 eV can be attributed to the Co (0) states in Co_3_Sn_2_S_2_ but is slightly higher than the value of metallic Co (778.1 eV) (*38*), further demonstrating the partial positive charge. A close investigation of the Co (0) 3*d*_3/2_ peak reveals an asymmetric line shape and a small plasmonic energy loss structure, which are characteristics of a good metallic sample (*39*, *40*). This is consistent with the band structure calculations, suggesting that the Fermi surface is dominated by Co 3*d* states. The BE of 781.1 eV can be ascribed to Co^2+^, which is a result of surface oxidation or loss of coordination such as S vacancies. The clearly distinguished, broad peak centered at 785.2 eV is a satellite structure for Co^δ+^ and Co^2+^ (*39*). The Sn 3*d* spectra indicate that the main peak is derived from the Sn^2+^states (fig. S14). All these results suggest that the bulk single-crystal Co_3_Sn_2_S_2_ has a different electronic configuration from that of Ni_3_Sn_2_S_2_, more likely to be (Co^δ+^)_3_(Sn^2 − γ^)_2_(S^2−^)_2_, with the Co atoms being partially positively charged and Sn atoms having an average valence below 2. For direct determination of the surface atomic termination, a bulk Co_3_Sn_2_S_2_ single crystal was cleaved under ultrahigh vacuum conditions to expose the (001) surface. Scanning tunneling microscope (STM) images were collected in situ at 2 K and are shown in Fig. 4C. The cleaved surface exhibits a typical Kagome-lattice atomic structure, indicating a Co layer at the surface. Such a cleave is energetically favorable because it exposes the smallest surface areas, and the S─Sn bond can be easily broken because of the large bonding distance (2.86 Å) (table S3).

## DISCUSSION

The high-quality bulk single crystal with well-defined surface termination provides an ideal platform for understanding the surface catalytic process. Although the details of the mechanism of the O═O bond formation remain unresolved, there is a consensus that the initial discharge of hydroxide ions at a catalytically active center is the initial step. For transition metals such as Co, Ni, and Fe, the crystal field formed by different types of coordination results in distinct spin states and *e*_g_ filling. In octahedrally coordinated systems, the *e*_g_ orbital has a large overlap with the oxygen-related adsorbates, making electron transfer between the active sites and adsorbates more favorable. This results the *e*_g_ filling of surface active centers as an activity descriptor for OER and oxygen reduction reaction (*12*, *41*). For our Co_3_Sn_2_S_2_ single crystals, the Co atoms in the bulk are octahedrally coordinated and the *d* orbitals are split into threefold degenerate *t*_2g_ states lying lower in energy and two-fold *e*_g_ states at higher energy (Fig. 4D and fig. S15). The intermediate spin states of Co with a magnetic moment of 0.31 μ_B_/Co indicates that the *e*_g_ orbitals are half-filled. However, for the surface Co atoms, the cleaving between S and Sn leads to loss of coordination and further breaking of the degeneracy of the *t*_2g_ and *e*_g_ orbitals, as illustrated in Fig. 4D (*42*, *43*). The half-filled *d*_z^2^_ orbital points toward the *p* orbital of the adsorbed hydroxide ions, resulting from the interaction with the bridging O^2−^ via π-donation. This gives rise to the formation of σ-bonding between the Co atoms and the surface OH^−^ adsorbates and favors electron transfer between them (*12*, *14*, *27*). In addition, loss of coordination for the surface Co octahedral leads to the formation of a highly distorted fivefold coordinated square pyramids. The newly open coordination sites make the uptake of OH^−^ more favorable (*15*). Our theoretical investigations and previous angle-resolved photoelectron spectroscopy measurements confirm that the surface states derived by Co are topologically protected. Different from the spin degeneracy caused by fabricating elemental vacancies, defects, or doping, which are easily destroyed by breaking the surface symmetry, the unoccupied TSSs of Co provide robust active sites for oxygen evolution. On the basis of the reaction mechanism, the kinetics of OH^−^ bonding via the surface Co atoms are determined by theoretical studies. As shown in fig. S16, OH^−^ binds to Co atoms and is located above the center of the three Co atoms. The bonding distance is determined to be 2.00 Å, which is shorter than the original Co─Sn bonding distance of 2.67 Å. The isosurface plot of the transferred charge distribution for the slab of the Co octahedral and OH^−^ adsorbate is shown in Fig. 4E. It can be seen that the interfacial charge distributions on the Co atom are dumbbell-like, indicating a 3*d*_z^2^_ orbital order (*43*), while the charge distribution on Sn is strictly spherical. The total charge on Co^δ+^ is calculated to be 8.75, while this value for OH^−^ is 7.67, vividly confirming electron transfer through the Co─O bonding. We also observed the similar electron transfer behavior on other adsorbate such as hydrogen (fig. S17). As two of the most important reaction intermediates of many surface reactions such as hydrogen evolution, hydrogen reduction, oxygen reduction, and H_2_O_2_ electrochemical synthesis, our results indicate that one can efficiently control the surface reactions by carefully tailoring the robust surface states of topological phase catalysts.

In conclusion, we synthesized high-quality Co_3_Sn_2_S_2_ bulk single crystals with well-defined atomic surface termination. Our magnetic, electrical resistivity, electrochemical measurements, XPS, STM, and density functional theory calculations uncovered the local spin structure and ligand environment of the surface Co atoms. The results provide new insights into the understanding of the surface water oxidation process. First, the nontrivial surface states derived by Co atoms are robust to surface distortion and modification. They are located just above the Fermi energy and can accept electrons from the adsorbates. In addition, loss of coordination of the Co atoms provides new open sites for the uptake of OH^−^ species. This facilitates the electron transfer through coupling between the *e*_g_ orbital of Co and the O-*p*_σ_ orbital of OH^−^. The present study provides a promising strategy to create highly efficient and robust catalysts by using the surface states around the Fermi energy.

## MATERIALS AND METHODS

### Materials synthesis

Co_3_Sn_2_S_2_ polycrystals were obtained by mixing high-purity elements with desired stoichiometry. The polycrystalline samples of Co_3_Sn_2_S_2_ were sealed in a quartz tube with some iodine under a partial Argon pressure. The samples were heated to 1000°C over 6 hours and kept for 24 hours before being slowly cooled to 600°C over 7 days. Large single-crystalline ingots up to centimeter size were obtained at the bottom of the quartz tubes.

### Characterization

Single-crystal XRD measurements were performed using a Bruker D8 Venture diffractometer equipped with a Triumph monochromator and a Photon100 area detector, operating with Mo Kα radiation. HRTEM was performed on a large lamella fabricated by FIB. The longitudinal electrical resistance measurement was conducted using a standard four-probe method with the alternating current (AC) transport option in a physical property measurement system (PPMS). XPS spectra were carried on a ultra-high vacuum (UHV) surface analysis system equipped with a Scienta-200 hemispherical analyzer. The base pressure of the sample analysis chamber is 2 × 10^−10^ mbar. Magnetization measurements were carried out on oriented crystals with the magnetic field applied along the *a* axes on the vibrating sample magnetometer (MPMS 3, Quantum Design). For STM, Co_3_Sn_2_S_2_ single crystals were cleaved in situ at *T* < 20 K to expose a (001) surface. After cleaving, the samples are quickly transferred to the STM head and kept in ultrahigh vacuum (*p* < 3 × 10^−9^ Pa) and at low temperature (*T* = 2 K). The tunneling spectra were measured using tungsten tips acquired by the standard lock-in technique.

### Electrocatalytic characterization

The assessment of OER activities were performed on the Autolab PGSTAT302N with an impedance module electrochemistry workstation. An Ag/AgCl (3 M KCl) electrode was used as the reference electrode, and a graphite rod was used as the counter electrode. The bulk Co_3_Sn_2_S_2_ single crystal was attached to a Cu wire with silver paint and served as the working electrode and catalyst. The LSVs were recorded with a scan rate of 5 mV/s. The electrochemical impedance spectroscopy was carried out with a 10-mV AC potential from 20 kHz to 0.01 Hz for correcting the polarization curves. All potentials were referenced to a reverse hydrogen electrode.

## Supplementary Material

http://advances.sciencemag.org/cgi/content/full/5/8/eaaw9867/DC1

Download PDF

Data S1

Data S2

## SUPPLEMENTARY MATERIALS

Supplementary material for this article is available at http://advances.sciencemag.org/cgi/content/full/5/8/eaaw9867/DC1 Calculation details Single-crystal XRD measurements Fig. S1. Band structure of Co_3_Sn_2_S_2_ with different strength of SOC. Fig. S2. Surface band structure of Co_3_Sn_2_S_2_ with different strength of SOC. Fig. S3. The surface states contributed by S atoms, and Sn atoms, respectively. Fig. S4. The surface states of Co_3_Sn_2_S_2_ with S termination. Fig. S5. Polarization curves of a Cu wire with silver paint and Co_3_Sn_2_S_2_ crystal. Fig. S6. Stability test of the crushed single-crystal catalyst on Ni foam. Fig. S7. SEM image of the crystal. Fig. S8. EDS spectra of the Co_3_Sn_2_S_2_ single crystal. Fig. S9. Powder XRD measurement of the crushed single crystal. Fig. S10. Crystal structure of Co_3_Sn_2_S_2_ at 100 K. Fig. S11. TEM image of the single crystal prepared using the FIB technique. Fig. S12. ZFC/FC curves for the single crystal. Fig. S13. XPS survey spectrum of the bulk single crystal. Fig. S14. High-resolution XPS spectra of Sn 3*d*. Fig. S15. The Co atoms (red) in Co_3_Sn_2_S_2_ are octahedrally coordinated. Fig. S16. The adsorption position of OH group on the crystal surface. Fig. S17. The adsorption position of H atom on the Co_3_Sn_2_S_2_ single-crystal surface. Table S1. Crystallographic and refinement parameters of Co_3_Sn_2_S_2_. Table S2. Fractional atomic coordinates of the crystal. Table S3. Selected interatomic distances. Data S1. Crystallographic information file obtained at 100 K. Data S2. Crystallographic information file obtained at 300 K. References (*44*–*47*)

## References

[1] Jing Y., Heine T., Two-dimensional Pd_3_P_2_S_8_ semiconductors as photocatalysts for the solar-driven oxygen evolution reaction: A theoretical investigation. J. Mater. Chem. A 6, 23495–23501 (2018).

[2] Banerjee T., Haase F., Savasci G., Gottschling K., Ochsenfeld C., Lotsch B. V., Single-site photocatalytic H_2_ evolution from covalent organic frameworks with molecular cobaloxime co-catalysts. J. Am. Chem. Soc. 139, 16228–16234 (2017). 29022345 10.1021/jacs.7b07489 PMC5691321

[3] Jo N. H., Wu Y., Wang L.-L., Orth P. P., Downing S. S., Manni S., Mou D., Johnson D. D., Kaminski A., Bud'ko S. L., Canfield P. C., Extremely large magnetoresistance and Kohler's rule in PdSn_4_: A complete study of thermodynamic, transport, and band-structure properties. Phys. Rev. B 96, 165145 (2017).

[4] Hasan M. Z., Xu S.-Y., Belopolski I., Huang S.-M., Discovery of Weyl fermion semimetals and topological Fermi arc states. Annu. Rev. Condens. Matter Phys. 8, 289–309 (2017).

[5] Müchler L., Zhang H., Chadov S., Yan B., Casper F., Kübler J., Zhang S.-C., Felser C., Topological insulators from a chemist's perspective. Angew. Chem. Int. Ed. Engl. 51, 7221–7225 (2012). 22684869 10.1002/anie.201202480

[6] Chen H., Zhu W., Xiao D., Zhang Z., CO oxidation facilitated by robust surface states on Au-covered topological insulators. Phys. Rev. Lett. 107, 056804 (2011). 21867089 10.1103/PhysRevLett.107.056804

[7] Li J., Ma H., Xie Q., Feng S., Ullah S., Li R., Dong J., Li D., Li Y., Chen X.-Q., Topological quantum catalyst: Dirac nodal line states and a potential electrocatalyst of hydrogen evolution in the TiSi family. Sci. China Mater. 61, 23–29 (2017).

[8] Schoop L. M., Pielnhofer F., Lotsch B. V., Chemical principles of topological semimetals. Chem. Mater. 30, 3155–3176 (2018).

[9] Rajamathi C. R., Gupta U., Pal K., Kumar N., Yang H., Sun Y., Shekhar C., Yan B., Parkin S., Waghmare U. V., Felser C., Rao C. N. R., Photochemical water splitting by bismuth chalcogenide topological insulators. ChemPhysChem 18, 2322–2327 (2017). 28683188 10.1002/cphc.201700344

[10] Zhao Y., Yang K. R., Wang Z., Yan X., Cao S., Ye Y., Dong Q., Zhang X., Thorne J. E., Jin L., Materna K. L., Trimpalis A., Bai H., Fakra S. C., Zhong X., Wang P., Pan X., Guo J., Flytzani-Stephanopoulos M., Brudvig G. W., Batista V. S., Wang D., Stable iridium dinuclear heterogeneous catalysts supported on metal-oxide substrate for solar water oxidation. Proc. Natl. Acad. Sci. U.S.A. 115, 2902–2907 (2018). 29507243 10.1073/pnas.1722137115 PMC5866603

[11] Li W., He D., Sheehan S. W., He Y., Thorne J. E., Yao X., Brudvig G. W., Wang D., Comparison of heterogenized molecular and heterogeneous oxide catalysts for photoelectrochemical water oxidation. Energ. Environ. Sci. 9, 1794–1802 (2016).

[12] Suntivich J., May K. J., Gasteiger H. A., Goodenough J. B., Shao-Horn Y., A perovskite oxide optimized for oxygen evolution catalysis from molecular orbital principles. Science 334, 1383–1385 (2011). 22033519 10.1126/science.1212858

[13] Zhang W., Banerjee-Ghosh K., Tassinari F., Naaman R., Enhanced electrochemical water splitting with chiral molecule-coated Fe_3_O_4_ nanoparticles. ACS Energy Lett. 3, 2308–2313 (2018).

[14] Kim H., Park J., Park I., Jin K., Jerng S. E., Kim S. H., Nam K. T., Kang K., Coordination tuning of cobalt phosphates towards efficient water oxidation catalyst. Nat. Commun. 6, 8253 (2015). 26365091 10.1038/ncomms9253 PMC4579784

[15] Kim J., Yin X., Tsao K.-C., Fang S., Yang H., Ca_2_Mn_2_O_5_ as oxygen-deficient perovskite electrocatalyst for oxygen evolution reaction. J. Am. Chem. Soc. 136, 14646–14649 (2014). 25295698 10.1021/ja506254g

[16] Tong Y., Guo Y., Chen P., Liu H., Zhang M., Zhang L., Yan W., Chu W., Wu C., Xie Y., Spin-state regulation of perovskite cobaltite to realize enhanced oxygen evolution activity. Chem 3, 812–821 (2017).

[17] Kim N.-I., Sa Y. J., Yoo T. S., Choi S. R., Afzal R. A., Choi T., Seo Y.-S., Lee K.-S., Hwang J. Y., Choi W. S., Joo S. H., Park J.-Y., Oxygen-deficient triple perovskites as highly active and durable bifunctional electrocatalysts for oxygen electrode reactions. Sci. Adv. 4, eaap9360 (2018). 29951583 10.1126/sciadv.aap9360 PMC6018999

[18] Liu E., Sun Y., Kumar N., Müchler L., Sun A., Jiao L., Yang S.-Y., Liu D., Liang A., Xu Q., Kroder J., Süß V., Borrmann H., Shekhar C., Wang Z., Xi C., Wang W., Schnelle W., Wirth S., Chen Y., Goennenwein S. T. B., Felser C., Giant anomalous Hall effect in a ferromagnetic Kagomé-lattice semimetal. Nat. Phys. 14, 1125–1131 (2018). 30416534 10.1038/s41567-018-0234-5 PMC6217931

[19] Xu Q., Liu E., Shi W., Muechler L., Gayles J., Felser C., Sun Y., Topological surface Fermi arcs in the magnetic Weyl semimetal Co_3_Sn_2_S_2_. Phys. Rev. B 97, 235416 (2018).

[20] Tripkovic V., Hansen H. A., Vegge T., From 3D to 2D Co and Ni oxyhydroxide catalysts: Elucidation of the active site and influence of doping on the oxygen evolution activity. ACS Catal. 7, 8558–8571 (2017).

[21] Yu M., Chan C. K., Tüysüz H., Coffee-waste templating of metal ion-substituted cobalt oxides for the oxygen evolution reaction. ChemSusChem 11, 605–611 (2018). 29194977 10.1002/cssc.201701877

[22] Wang X., Yu L., Guan B. Y., Song S., Lou X. W., Metal–organic framework hybrid-assisted formation of Co_3_O_4_ /Co-Fe oxide double-shelled nanoboxes for enhanced oxygen evolution. Adv. Mater. 30, 1801211 (2018). 10.1002/adma.201801211 29782694

[23] Kassem M. A., Tabata Y., Waki T., Nakamura H., Low-field anomalous magnetic phase in the kagome-lattice shandite Co_3_Sn_2_S_2_. Phy. Rev. B 96, 014429 (2017).

[24] Xia C., Jiang Q., Zhao C., Hedhili M. N., Alshareef H. N., Selenide-based electrocatalysts and scaffolds for water oxidation applications. Adv. Mater. 28, 77–85 (2016). 26540620 10.1002/adma.201503906

[25] Zhang Y., Ouyang B., Xu J., Jia G., Chen S., Rawat R. S., Fan H. J., Rapid synthesis of cobalt nitride nanowires: Highly efficient and low-cost catalysts for oxygen evolution. Angew. Chem. Int. Ed. Engl. 55, 8670–8674 (2016). 27254484 10.1002/anie.201604372

[26] Menezes P. W., Panda C., Garai S., Walter C., Guiet A., Driess M., Structurally ordered intermetallic cobalt stannide nanocrystals for high-performance electrocatalytic overall water-splitting. Angew. Chem. Int. Ed. Engl. 57, 15237–15242 (2018). 30248219 10.1002/anie.201809787

[27] Zhao S., Wang Y., Dong J., He C.-T., Yin H., An P., Zhao K., Zhang X., Gao C., Zhang L., Lv J., Wang J., Zhang J., Khattak A. M., Khan N. A., Wei Z., Zhang J., Liu S., Zhao H., Tang Z., Ultrathin metal–organic framework nanosheets for electrocatalytic oxygen evolution. Nat. Energy 1, 16184 (2016).

[28] Liu Y., Cheng H., Lyu M., Fan S., Liu Q., Zhang W., Zhi Y., Wang C., Xiao C., Wei S., Ye B., Xie Y., Low overpotential in vacancy-rich ultrathin CoSe_2_ nanosheets for water oxidation. J. Am. Chem. Soc. 136, 15670–15675 (2014). 25310506 10.1021/ja5085157

[29] Chen P., Xu K., Fang Z., Tong Y., Wu J., Lu X., Peng X., Ding H., Wu C., Xie Y., Metallic Co_4_N porous nanowire arrays activated by surface oxidation as electrocatalysts for the oxygen evolution reaction. Angew. Chem. Int. Ed. Engl. 54, 14710–14714 (2015). 26437900 10.1002/anie.201506480

[30] Pandey K., Islam S. T. A., Happe T., Armstrong F. A., Frequency and potential dependence of reversible electrocatalytic hydrogen interconversion by [FeFe]-hydrogenases. Proc. Natl. Acad. Sci. U.S.A. 114, 3843–3848 (2017). 28348243 10.1073/pnas.1619961114 PMC5393252

[31] Shinagawa T., Garcia-Esparza A. T., Takanabe K., Insight on Tafel slopes from a microkinetic analysis of aqueous electrocatalysis for energy conversion. Sci. Rep. 5, 13801 (2015). 26348156 10.1038/srep13801 PMC4642571

[32] Damjanovic A., Dey A., Bockris J. O., Kinetics of oxygen evolution and dissolution on platinum electrodes. Electrochim. Acta 11, 791–814 (1966).

[33] Hou Y., Lohe M. R., Zhang J., Liu S., Zhuang X., Feng X., Vertically oriented cobalt selenide/NiFe layered-double-hydroxide nanosheets supported on exfoliated graphene foil: An efficient 3D electrode for overall water splitting. Energ. Environ. Sci. 9, 478–483 (2016).

[34] Li G., Sun Y., Rao J., Wu J., Kumar A., Xu Q., Fu C., Liu E., Blake R. G., Werner P., Shao B., Liu K., Parkin S., Liu X., Fahlman M., Liou S.-C., Auffermann G., Zhang J., Felser C., Feng X., Carbon-tailored semimetal MoP as an efficient hydrogen evolution electrocatalyst in both alkaline and acid media. Adv. Energ. Mater. 8, 1801258 (2018).

[35] Cummins D. R., Martinez U., Sherehiy A., Kappera R., Martinez-Garcia A., Schulze R. K., Jasinski J., Zhang J., Gupta R. K., Lou J., Chhowalla M., Sumanasekera G., Mohite A. D., Sunkara M. K., Gupta G., Efficient hydrogen evolution in transition metal dichalcogenides via a simple one-step hydrazine reaction. Nat. Commun. 7, 11857 (2016). 27282871 10.1038/ncomms11857 PMC4906413

[36] Gütlich P., Range K. J., Felser C., Schultz-Münzenberg C., Tremel W., Walcher D., Waldeck M., The valence states of nickel, tin, and sulfur in the ternary chalcogenide Ni_3_Sn_2_S_2_—XPS, ^61^Ni and ^119^Sn Mössbauer investigations, and band structure calculations. Angew. Chem. Int. Ed. 38, 2381–2384 (1999). 10.1002/(sici)1521-3773(19990816)38:16<2381::aid-anie2381>3.0.co;2-l 10458793

[37] Holder M., Dedkov Y. S., Kade A., Rosner H., Schnelle W., Leithe-Jasper A., Weihrich R., Molodtsov S. L., Photoemission study of electronic structure of the half-metallic ferromagnet Co_3_Sn_2_S_2_. Phys. Rev. B 79, 205116 (2009).

[38] Biesinger M. C., Payne B. P., Grosvenor A. P., Lau L. W. M., Gerson A. R., Smart R. S. C., Resolving surface chemical states in XPS analysis of first row transition metals, oxides and hydroxides: Cr, Mn, Fe, Co and Ni. Appl. Surf. Sci. 257, 2717–2730 (2011).

[39] Grosvenor A. P., Wik S. D., Cavell R. G., Mar A., Examination of the bonding in binary transition-metal monophosphides MP (M = Cr, Mn, Fe, Co) by X-ray photoelectron spectroscopy. Inorg. Chem. 44, 8988–8998 (2005). 16296854 10.1021/ic051004d

[40] Noh H.-J., Jeong J., Jeong J., Cho E.-J., Kim S. B., Kim K., Min B. I., Kim H.-D., Anisotropic electric conductivity of delafossite PdCoO_2_ studied by angle-resolved photoemission spectroscopy. Phys. Rev. Lett. 102, 256404 (2009). 19659104 10.1103/PhysRevLett.102.256404

[41] Suntivich J., Gasteiger H. A., Yabuuchi N., Nakanishi H., Goodenough J. B., Shao-Horn Y., Design principles for oxygen-reduction activity on perovskite oxide catalysts for fuel cells and metal–air batteries. Nat. Chem. 3, 546–550 (2011). 21697876 10.1038/nchem.1069

[42] Liu Y., Yin S., Shen P. K., Asymmetric 3d electronic structure for enhanced oxygen evolution catalysis. ACS Appl. Mater. Interfaces 10, 23131–23139 (2018). 29916699 10.1021/acsami.8b06106

[43] Lee J., Lin C., Demkov A. A., Metal-induced charge transfer, structural distortion, and orbital order in SrTiO_3_ thin films. Phys. Rev. B 87, 165103 (2013).

[44] Kresse G., Furthmüller J., Efficient iterative schemes for *ab initio* total-energy calculations using a plane-wave basis set. Phys. Rev. B 54, 11169–11186 (1996). 10.1103/physrevb.54.11169 9984901

[45] Perdew J. P., Burke K., Ernzerhof M., Generalized gradient approximation made simple. Phys.Rev. Lett. 77, 3865–3868 (1996). 10062328 10.1103/PhysRevLett.77.3865

[46] Mostofi A. A., Yates J. R., Lee Y.-S., Souza I., Vanderbilt D., Marzari N., wannier90: A tool for obtaining maximally-localised Wannier functions. Comput. Phys. Commun. 178, 685–699 (2008).

[47] Weng H., Dai X., Fang Z., Exploration and prediction of topological electronic materials based on first-principles calculations. MRS Bull. 39, 849–858 (2014).

