# Supplementary material for: Surface states in bulk single crystal of topological semimetal Co3Sn2S2 toward water oxidation
Source: Sci Adv. 2019 Aug 16;5(8):eaaw9867. doi: 10.1126/sciadv.aaw9867 (PMC6697436; doi:10.1126/sciadv.aaw9867)
Supplement: http://advances.sciencemag.org/cgi/content/full/5/8/eaaw9867/DC1 [file supp_5_8_eaaw9867__index.html]

Science Advances | Science AdvancesAAASSearchScience AdvancesMenu

## Supplementary Materials

**The PDF file includes:**

- Calculation details
- Single-crystal XRD measurements
- Fig. S1. Band structure of Co3Sn2S2 with different strength of SOC.
- Fig. S2. Surface band structure of Co3Sn2S2 with different strength of SOC.
- Fig. S3. The surface states contributed by S atoms, and Sn atoms, respectively.
- Fig. S4. The surface states of Co3Sn2S2 with S termination.
- Fig. S5. Polarization curves of a Cu wire with silver paint and Co3Sn2S2 crystal.
- Fig. S6. Stability test of the crushed single-crystal catalyst on Ni foam.
- Fig. S7. SEM image of the crystal.
- Fig. S8. EDS spectra of the Co3Sn2S2 single crystal.
- Fig. S9. Powder XRD measurement of the crushed single crystal.
- Fig. S10. Crystal structure of Co3Sn2S2 at 100 K.
- Fig. S11. TEM image of the single crystal prepared using the FIB technique.
- Fig. S12. ZFC/FC curves for the single crystal.
- Fig. S13. XPS survey spectrum of the bulk single crystal.
- Fig. S14. High-resolution XPS spectra of Sn 3*d*.
- Fig. S15. The Co atoms (red) in Co3Sn2S2 are octahedrally coordinated.
- Fig. S16. The adsorption position of OH group on the crystal surface.
- Fig. S17. The adsorption position of H atom on the Co3Sn2S2 single-crystal surface.
- Table S1. Crystallographic and refinement parameters of Co3Sn2S2.
- Table S2. Fractional atomic coordinates of the crystal.
- Table S3. Selected interatomic distances.
- References (*44*–*47*)

Download PDF

**Other Supplementary Material for this manuscript includes the following:**

- Data S1 (.cif file). Crystallographic information file obtained at 100 K.
- Data S2 (.cif file). Crystallographic information file obtained at 300 K.

**Files in this Data Supplement:**

- Adobe PDF - aaw9867\_SM.pdf
