# Supplementary material for: Surface states in bulk single crystal of topological semimetal Co3Sn2S2 toward water oxidation
Source: Sci Adv. 2019 Aug 16;5(8):eaaw9867. doi: 10.1126/sciadv.aaw9867 (PMC6697436; doi:10.1126/sciadv.aaw9867)
Supplement: Download PDF [file aaw9867_SM.pdf]

Science Advances
AAAS

advances.sciencemag.org/cgi/content/full/5/8/eaaw9867/DC1

Supplementary Materials for

Surface states in bulk single crystal of topological semimetal $Co_3Sn_2S_2$ toward water oxidation

Guowei Li*, Qiunan Xu, Wujun Shi, Chenguang Fu, Lin Jiao, Machteld E. Kamminga, Mingquan Yu, Harun Tüysüz, Nitesh Kumar, Vicky Süß, Rana Saha, Abhay K. Srivastava, Steffen Wirth, Gudrun Auffermann, Johannes Gooth, Stuart Parkin, Yan Sun*, Enke Liu*, Claudia Felser*

*Corresponding author. Email: guowei.li@cpfs.mpg.de (G.L.); yan.sun@cpfs.mpg.de (Y.S.); ekliu@iphy.ac.cn (E.L.); claudia.felser@cpfs.mpg.de (C.F.)

Published 16 August 2019, *Sci. Adv.* **5**, eaaw9867 (2019)
DOI: 10.1126/sciadv.aaw9867

The PDF file includes:

Calculation details
Single-crystal XRD measurements
Fig. S1. Band structure of $Co_3Sn_2S_2$ with different strength of SOC.
Fig. S2. Surface band structure of $Co_3Sn_2S_2$ with different strength of SOC.
Fig. S3. The surface states contributed by S atoms, and Sn atoms, respectively.
Fig. S4. The surface states of $Co_3Sn_2S_2$ with S termination.
Fig. S5. Polarization curves of a Cu wire with silver paint and $Co_3Sn_2S_2$ crystal.
Fig. S6. Stability test of the crushed single-crystal catalyst on Ni foam.
Fig. S7. SEM image of the crystal.
Fig. S8. EDS spectra of the $Co_3Sn_2S_2$ single crystal.
Fig. S9. Powder XRD measurement of the crushed single crystal.
Fig. S10. Crystal structure of $Co_3Sn_2S_2$ at 100 K.
Fig. S11. TEM image of the single crystal prepared using the FIB technique.
Fig. S12. ZFC/FC curves for the single crystal.
Fig. S13. XPS survey spectrum of the bulk single crystal.
Fig. S14. High-resolution XPS spectra of Sn 3*d*.
Fig. S15. The Co atoms (red) in $Co_3Sn_2S_2$ are octahedrally coordinated.
Fig. S16. The adsorption position of OH group on the crystal surface.
Fig. S17. The adsorption position of H atom on the $Co_3Sn_2S_2$ single-crystal surface.
Table S1. Crystallographic and refinement parameters of $Co_3Sn_2S_2$.
Table S2. Fractional atomic coordinates of the crystal.
Table S3. Selected interatomic distances.
References (*44–47*)

Other Supplementary Material for this manuscript includes the following:

(available at advances.sciencemag.org/cgi/content/full/5/8/eaaw9867/DC1)

Data S1 (.cif file). Crystallographic information file obtained at 100 K.
Data S2 (.cif file). Crystallographic information file obtained at 300 K.

Calculation details

To explore the topological prosperities of the paramagnetic $Co_3Sn_2S_2$, we use the Vienna Ab initio Simulation Package (VASP) (*44*) based on density functional theory (DFT) to perform the first-principles calculations. The exchange-correlation potential that we choose here is the generalized gradient approximation of the Perdew-Burke-Ernzerhof functional (*45*) and spin–orbital coupling (SOC) is included. After OH or H adsorption, the OH or H groups are fully relaxed until the force less than 10 meV / Å. The energy cutoff is set as 300 eV. The k-points grid is 6 x 6 x 1. Moreover, we also construct a tight-binding Hamiltonian from the maximally localized Wannier functions (MLWFs) (*46*) overlap matrix which is derived from the Co 3d, Sn 5p, and Se 4p orbitals, and the surface states for a semi-infinite slab along the z direction can be calculated according to this Hamiltonian by Green's function method (*47*).

Density functional theory (DFT) method as implemented in the Vienna ab initio Simulation Package (VASP) was used for the electronic structure calculations. The exchange–correlation was considered in the revised Perdew-Burke-Ernzerhof (rPBE) parameterized generalized gradient approximation (GGA) and spin–orbital coupling (SOC) was included. After OH (H) adsorption, the OH (H) groups are fully relaxed until the force less than 10 meV / Å. The energy cutoff is set as 300 eV. The k-points grid is 6 x 6 x 1.

Single-crystal XRD measurements

Single crystal XRD measurements were performed using a Bruker D8 Venture diffractometer equipped with a Triumph monochromator and a Photon100 area detector, operating with Mo Kα radiation. The crystals were mounted on a 0.2 mm nylon loop using cryo-oil. The crystals were cooled with a nitrogen flow from an Oxford Cryosystems Plus. Data processing was done using the Bruker Apex III software, the structures were solved using direct methods and the SHELX97 software was used for structure refinement.

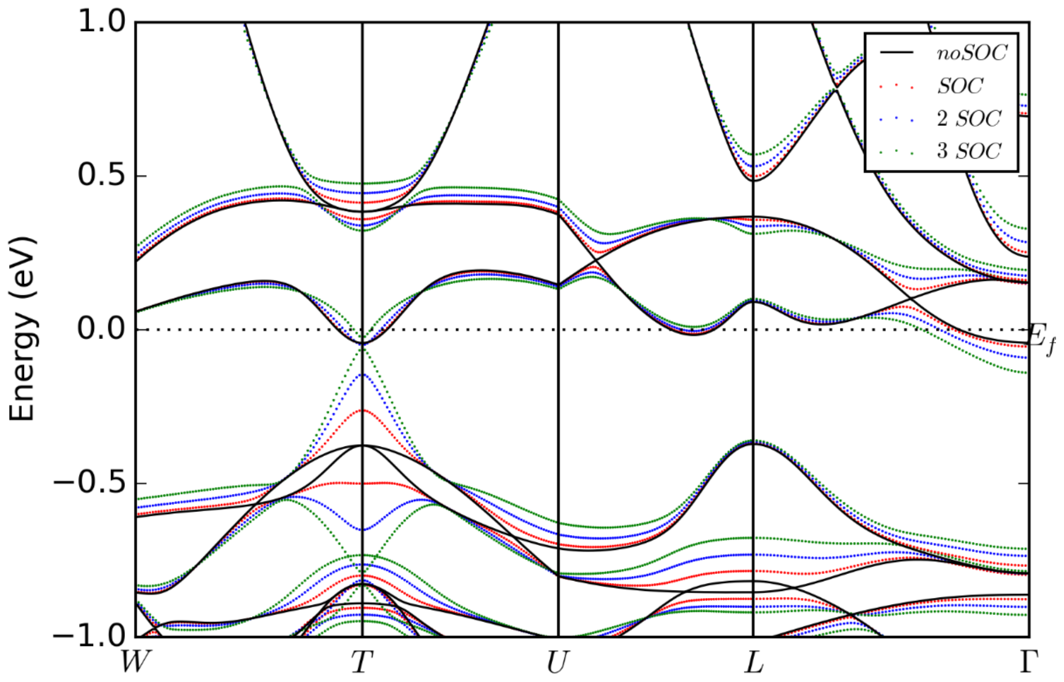

Fig. S1. Band structure of $Co_3Sn_2S_2$ with different strength of SOC. The band gap is strongly depended on the strength of the SOC effect.

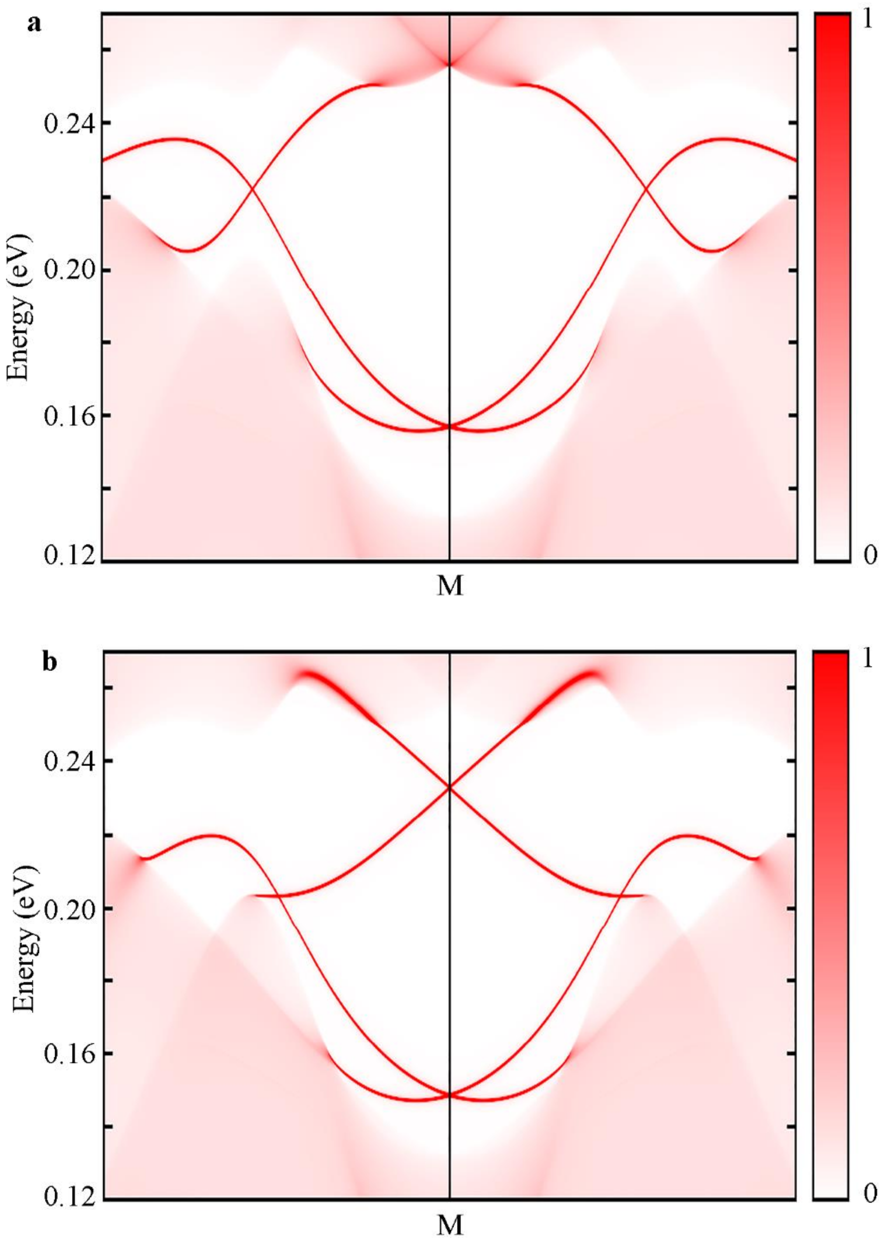

Fig. S2. Surface band structure of $Co_3Sn_2S_2$ with different strength of SOC. a. 2 SOC, and **b.** 3 SOC. The linear crossing can be observed clearly, with the crossing point sitting just 0.23 eV above the Fermi level.

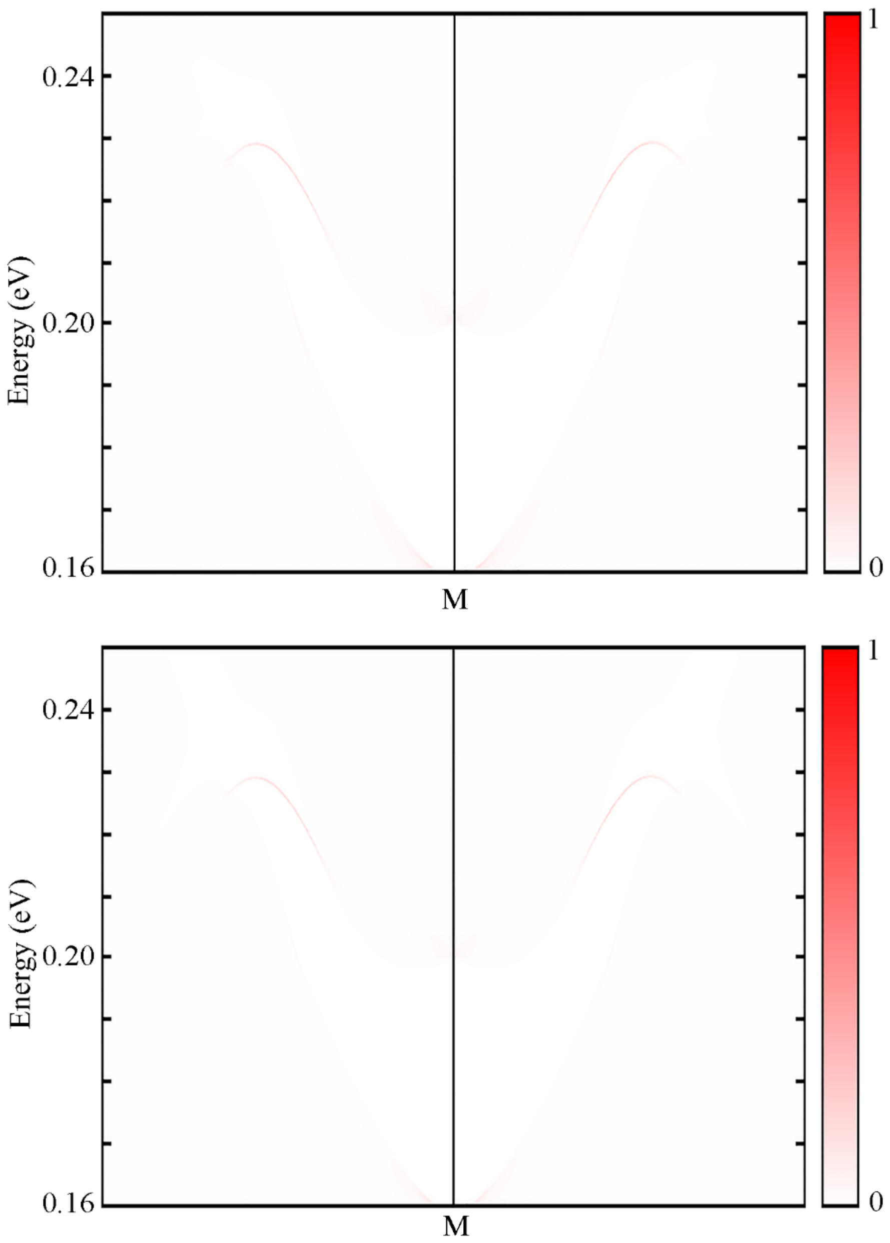

Fig. S3. The surface states contributed by S atoms, and Sn atoms, respectively. The surface states contributed by **a.** S atoms, and **b.** Sn atoms, respectively. It can be concluded that the surface S and Sn atoms make negligible contributions to the surface states.

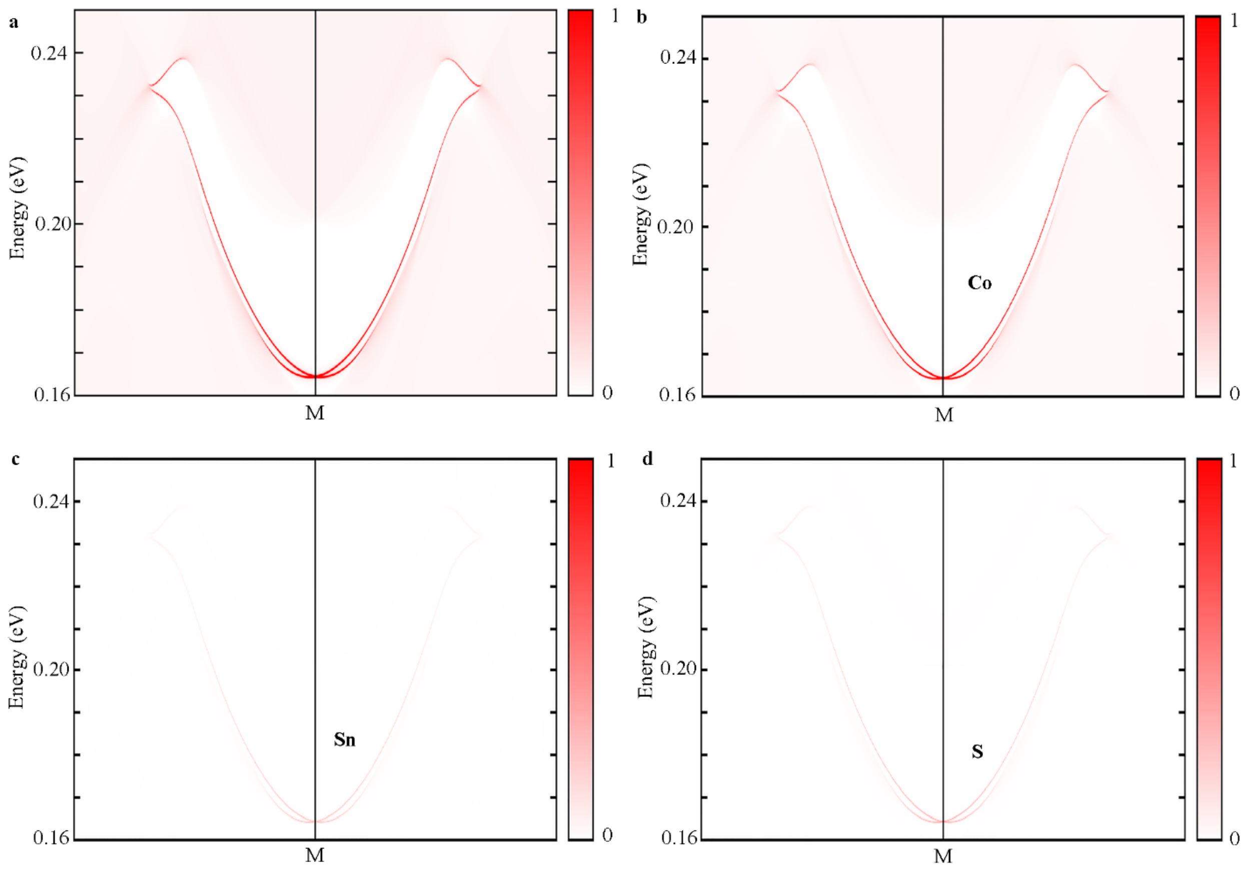

Fig. S4. The surface states of $Co_3Sn_2S_2$ with S termination. a. The surface states of $Co_3Sn_2S_2$ with S termination. The contributions to the surface states from **b.** Co atoms, **c.** Sn atoms, and **d.** S atoms. It can be seen clearly that the surface states are almost completely derived from the surface Co atoms.

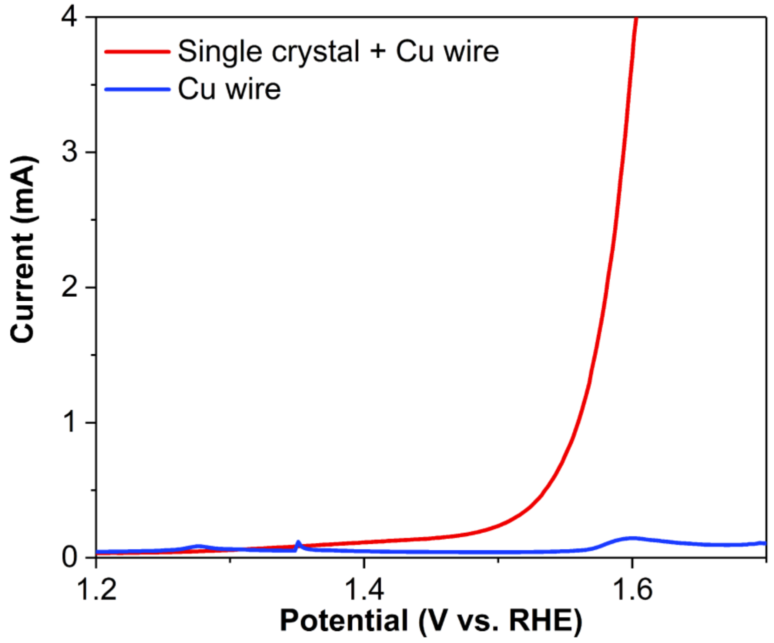

Fig. S5. Polarization curves of a Cu wire with silver paint and $Co_3Sn_2S_2$ crystal.

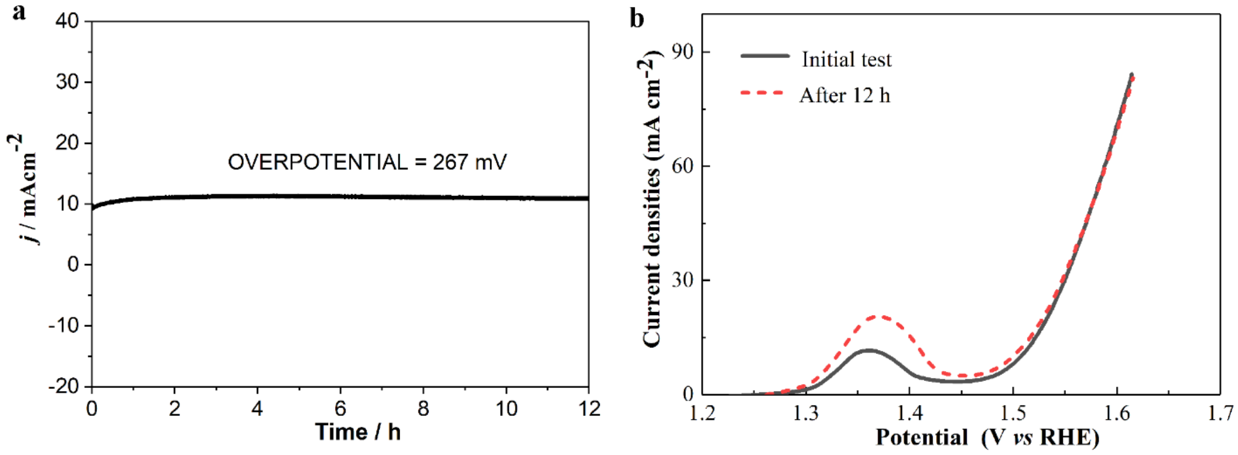

Fig. S6. Stability test of the crushed single-crystal catalyst on Ni foam. a. The durability test for 12 h reveals the high stability of the microcrystal catalyst. **b.** Polarization curves of the crushed single crystal on Ni foam before and after stability test.

Table S1. Crystallographic and refinement parameters of $Co_3Sn_2S_2$, measured at 100 K and 300 K.

$Co_3Sn_2S_2$		
Formula weight	478.29 g/mol	
Crystal size	0.28 × 0.18 × 0.10 mm^3	
Color	black	
Wavelength	0.71073 Å (Mo Kα radiation)	
Refinement method	full matrix least squares on F^2, anisotropic displacement parameters	
Absorption correction	multi-scan	
**Temperature**	**100 K**	**300 K**
crystal system	trigonal	trigonal
space group	*R*-3*m* (no. 166)	*R*-3*m* (no. 166)
symmetry	centrosymmetric	centrosymmetric
Z	3	3
D (calculated) (g/cm^3)	7.304	7.239
F(000)	639	639
a (Å)	5.3576(2)	5.3643(16)
b (Å)	5.3576(2)	5.3643(16)
c (Å)	13.1231(14)	13.208(4)
α (°)	90.0	90.0
β (°)	90.0	90.0
γ (°)	120.0	120.0
volume ($Å^3$)	326.22(4)	329.1(2)
absorption coefficient (mm^{-1})	23.280	23.073
min / max transmission factor	0.0099 / 0.0484	0.0089 / 0.0492
θ range (degrees)	4.66 – 35.73	4.63 – 36.31
index ranges	$-7 < h < 7$	$-7 < h < 7$
	$-7 < k < 7$	$-7 < k < 7$
	$-18 < l < 18$	$-18 < l < 18$
data / restraints / parameters	145 / 0 / 13	149 / 0 / 13
GooF on F^2	1.442	1.311
no. total reflections	2977	4699
no. unique reflections	145	149
no. obs Fo > 4σ(Fo)	145	149
R_1 [Fo > 4σ(Fo)]	0.0235	0.0312
R_1 [all data]	0.0235	0.0312
wR_2 [Fo > 4σ(Fo)]	0.0548	0.0680
wR_2 [all data]	0.0548	0.0680
largest peak and hole ($eÅ^{-3}$)	1.45 and -0.90	2.35 and -2.99

Table S2. Fractional atomic coordinates of the crystal.

100 K

Atom	*x*	*y*	*z*	U_{eq} (Å^2)
Co1	0.50000	0.00000	0.50000	0.0039(5)
Sn1	0.00000	0.00000	0.00000	0.0052(5)
Sn2	0.00000	0.00000	0.50000	0.0052(4)
S1	0.00000	0.00000	0.28276(19)	0.0034(6)

300 K

Atom	*x*	*y*	*z*	U_{eq} (Å^2)
Co1	0.50000	0.00000	0.50000	0.0116(5)
Sn1	0.00000	0.00000	0.00000	0.0117(4)
Sn2	0.00000	0.00000	0.50000	0.0125(4)
S1	0.00000	0.00000	0.28312(18)	0.0138(6)

Table S3. Selected interatomic distances.

100 K		
Interatomic distance (Å)		
Co	Sn	2.6787(2)
Co	S	2.1743(16)
Sn	S	2.864(3)

300 K		
Interatomic distance (Å)		
Co	Sn	2.6822 (8)
Co	S	2.1793(18)
Sn	S	2.869(3)

Associated content

Supporting Information includes crystallographic information files of $Co_3Sn_2S_2$, measured at 100 K and 300 K (CIF).

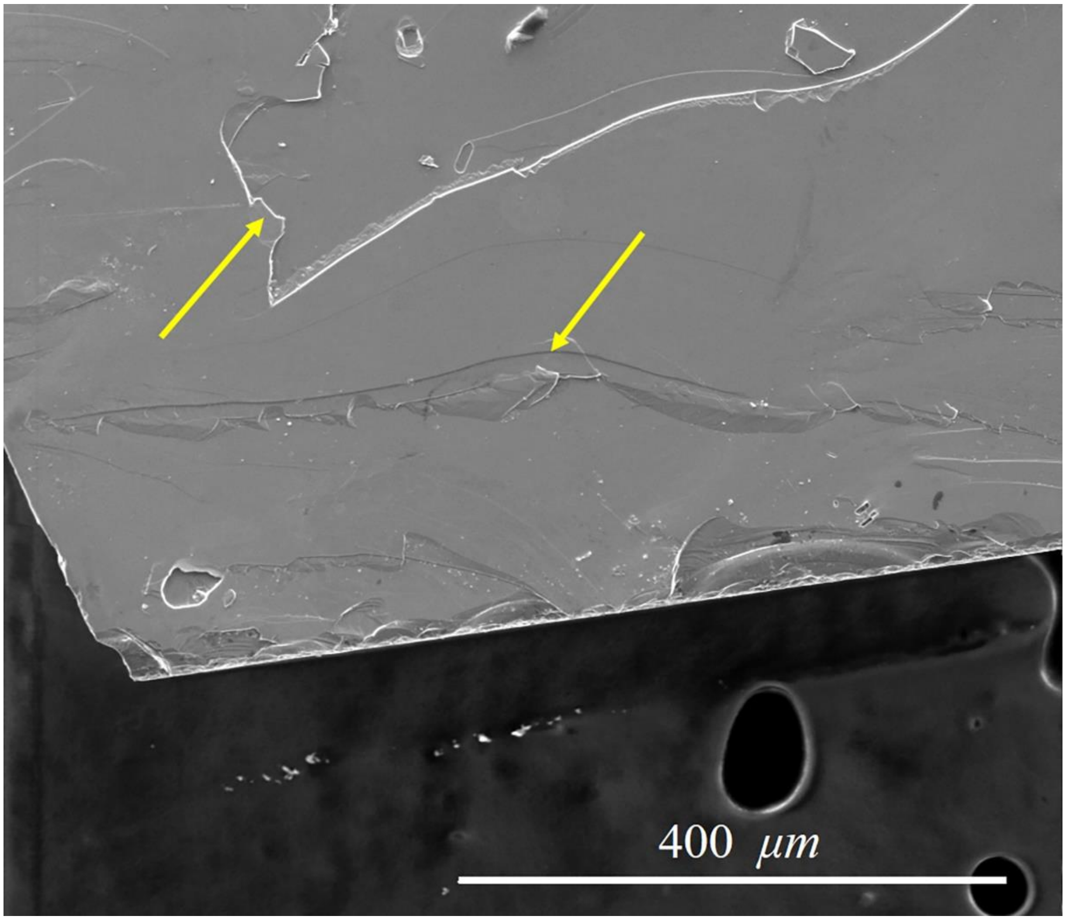

Fig. S7. SEM image of the crystal. SEM image of the crystal surface, which shows the layer nature of the crystal. The layer nature is showed by yellow arrows.

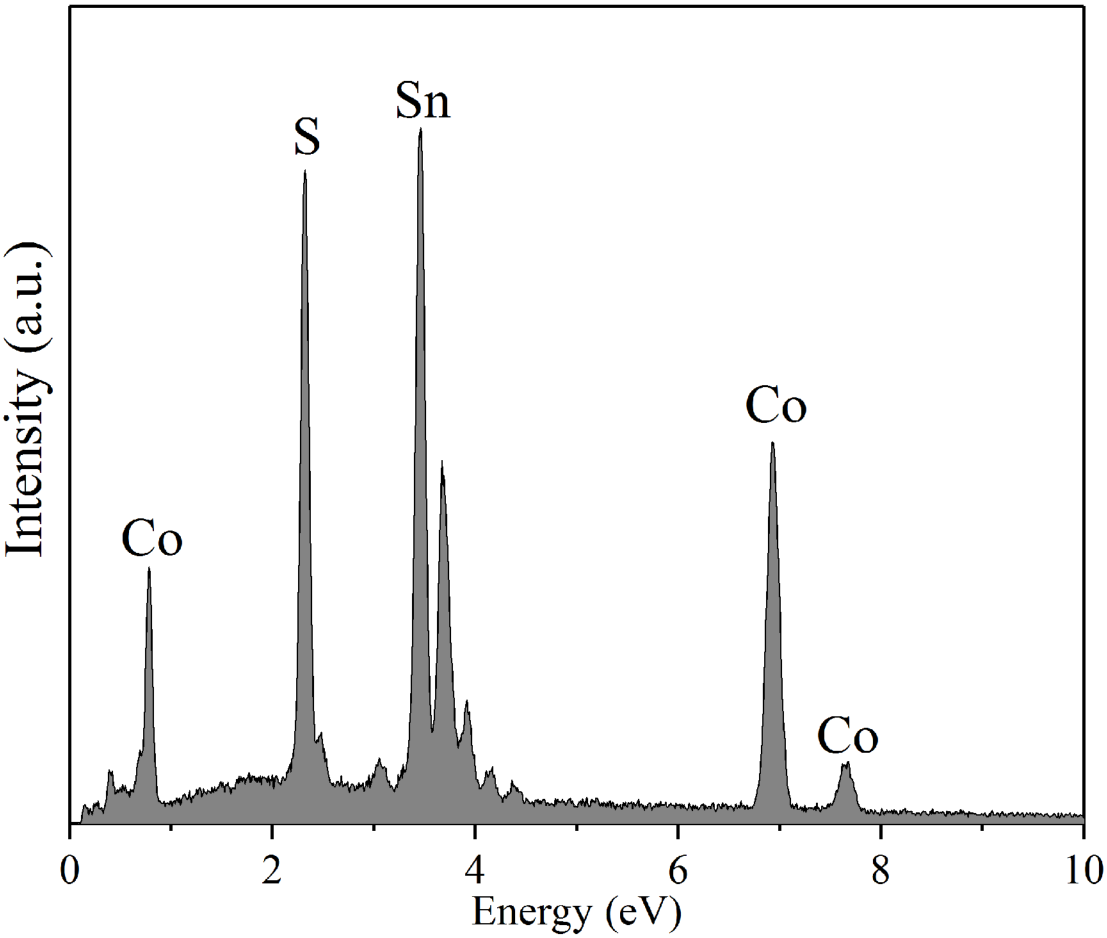

Fig. S8. EDS spectra of the $Co_3Sn_2S_2$ single crystal. Only Co, S, and Sn signals are observed. The molar ratio between these elements are consistent with the formula of $Co_3Sn_2S_2$.

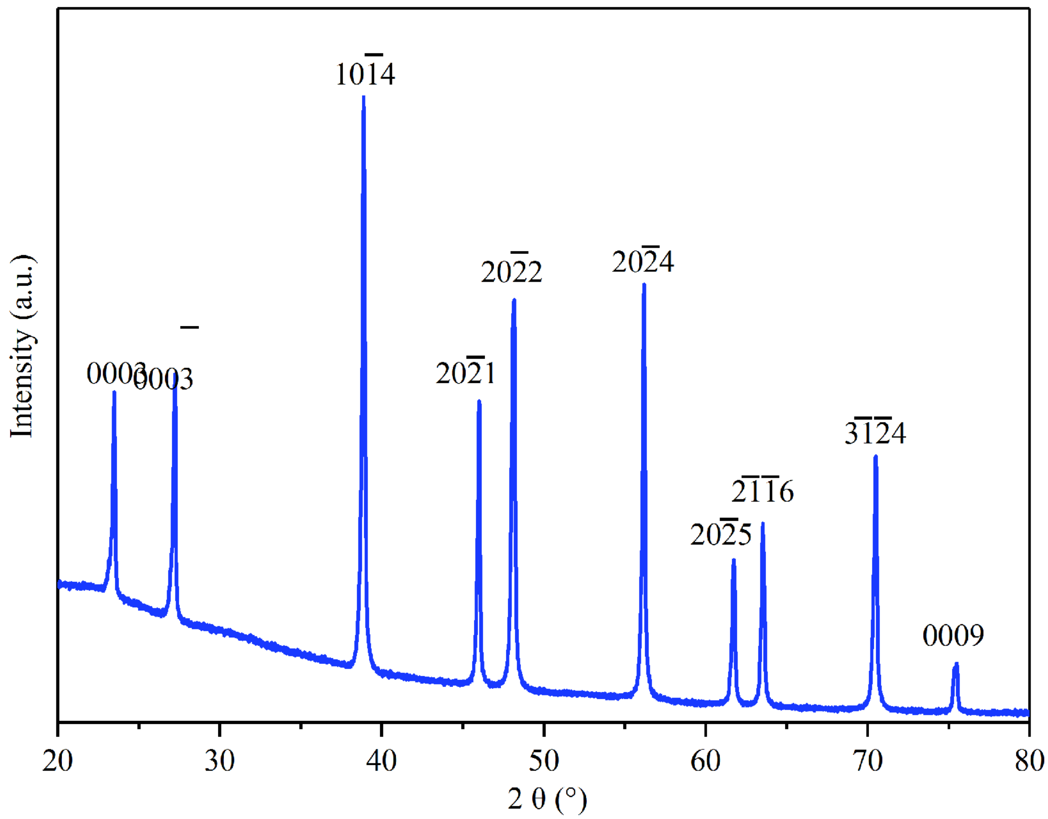

Fig. S9. Powder XRD measurement of the crushed single crystal. No extra peaks are found for metal oxides, or other impurities.

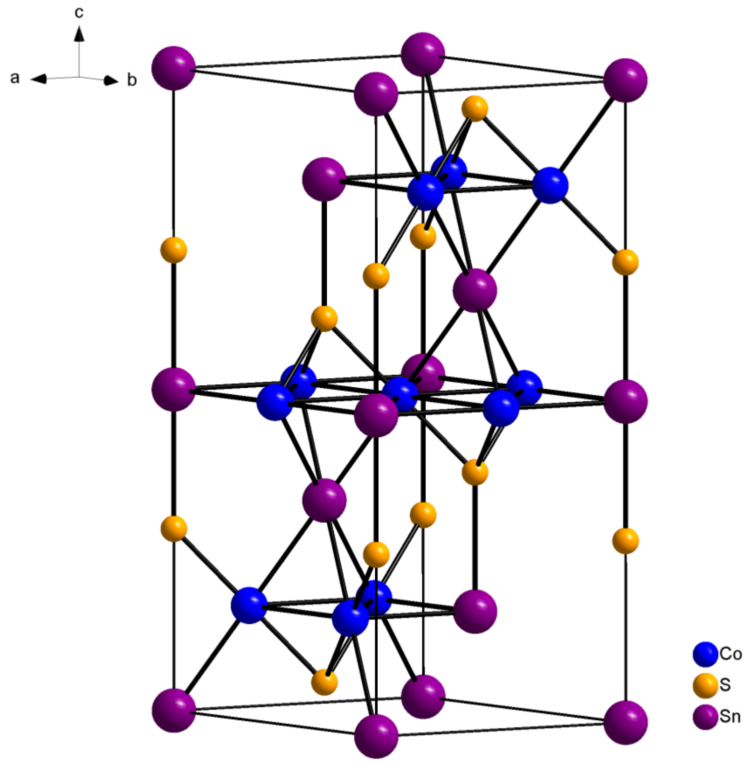

Fig. S10. Crystal structure of $Co_3Sn_2S_2$ at 100 K, derived from the single crystal XRD data.

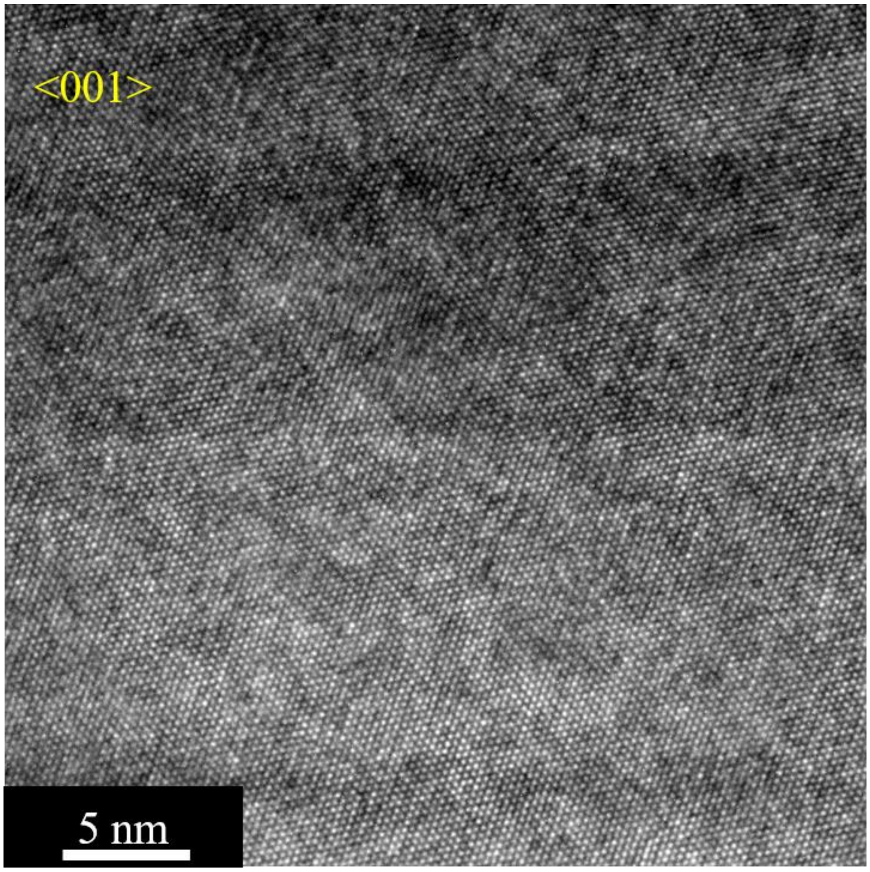

Fig. S11. TEM image of the single crystal prepared using the FIB technique. The clearly and sharp lattice structure indicates the high crystalline quality and purity of the $Co_3Sn_2S_2$ single crystal.

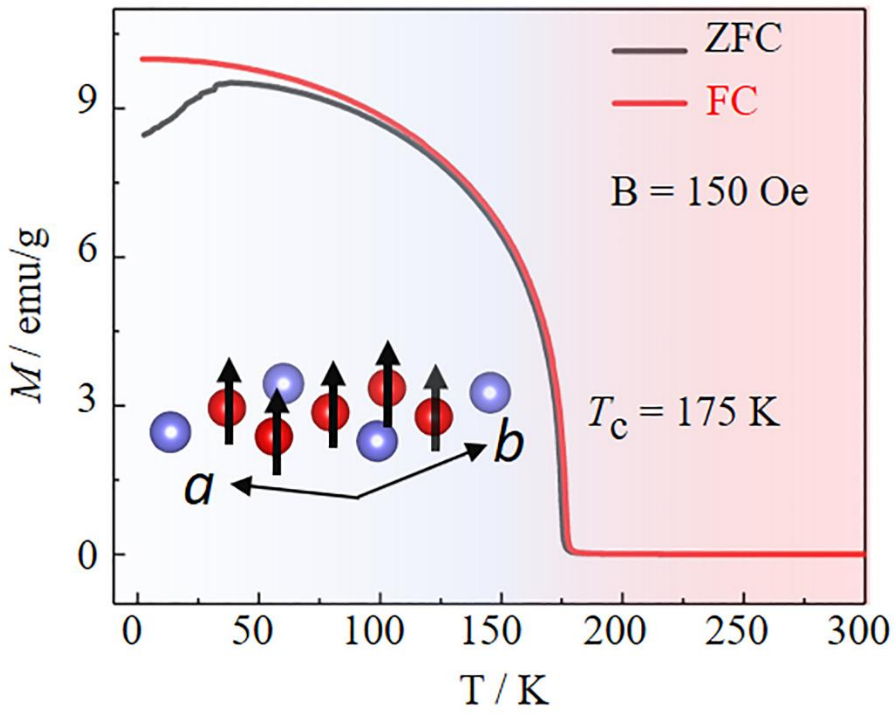

Fig. S12. ZFC/FC curves for the single crystal. The magnetic field was applied along the *c* axis. The Curie temperature (T_C) is estimated to 175 K. The magnetic moments are derived from Co atoms in the Kagome lattice.

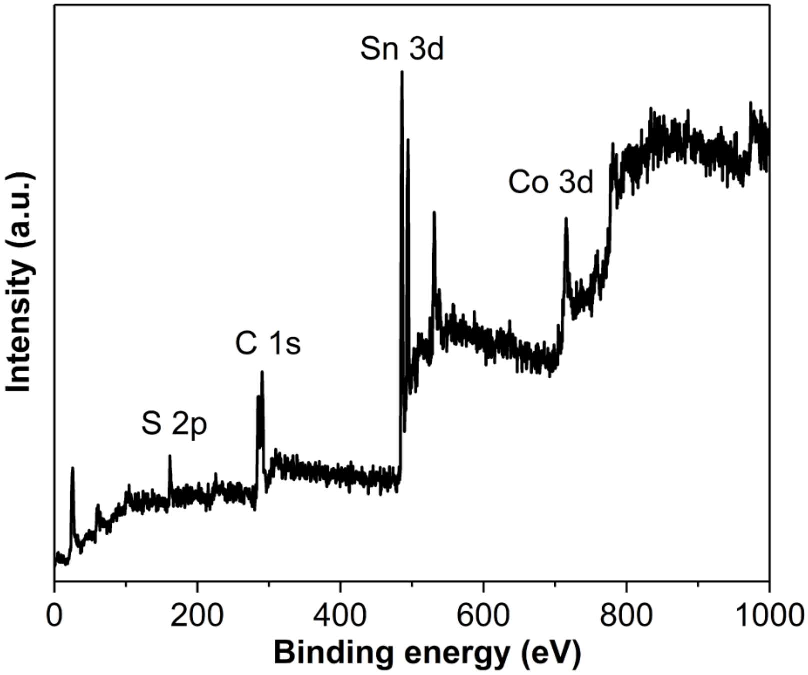

Fig. S13. XPS survey spectrum of the bulk single crystal.

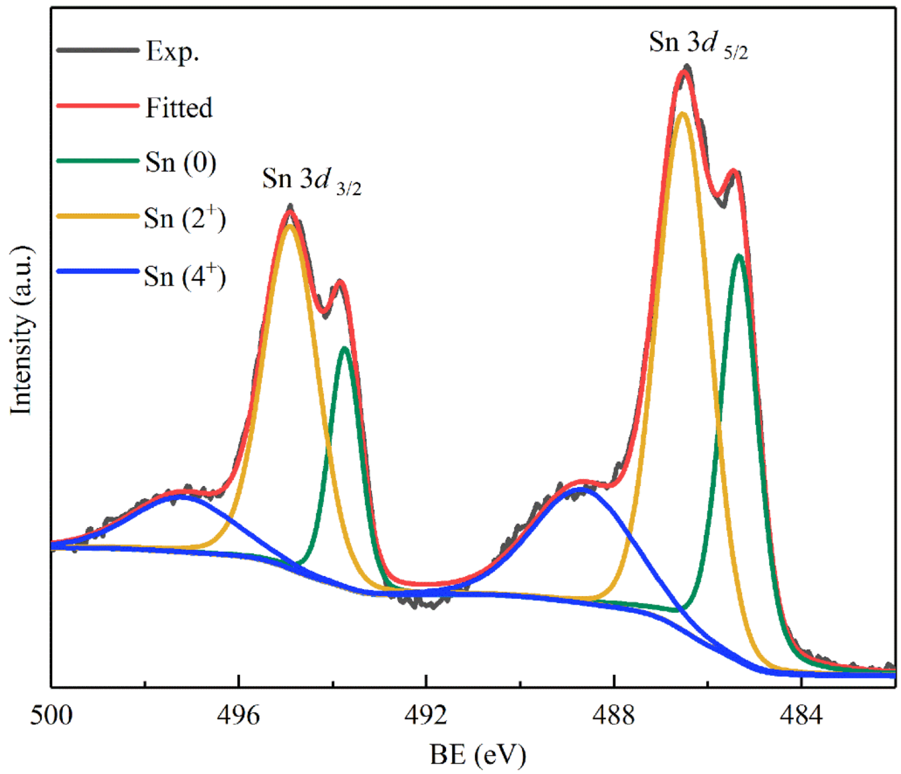

Fig. S14. High-resolution XPS spectra of Sn 3*d*.

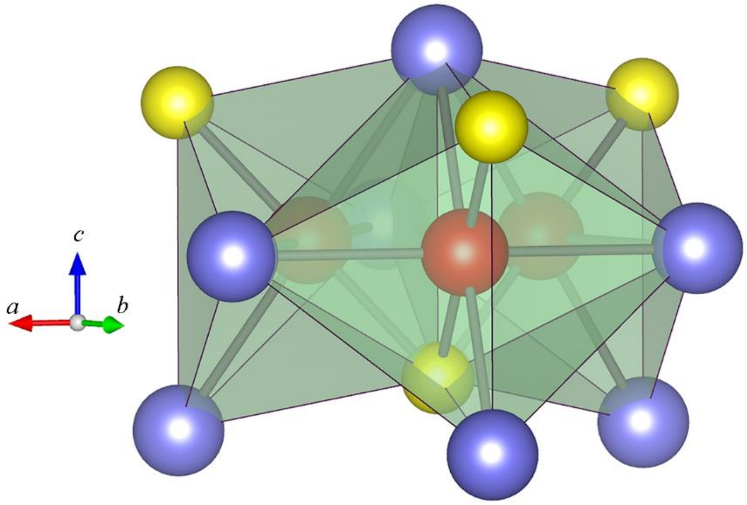

Fig. S15. The Co atoms (red) in $Co_3Sn_2S_2$ are octahedrally coordinated. Four Sn atoms (blue) are in the same plane, while two S atoms (yellow) are sitting at the top and down position.

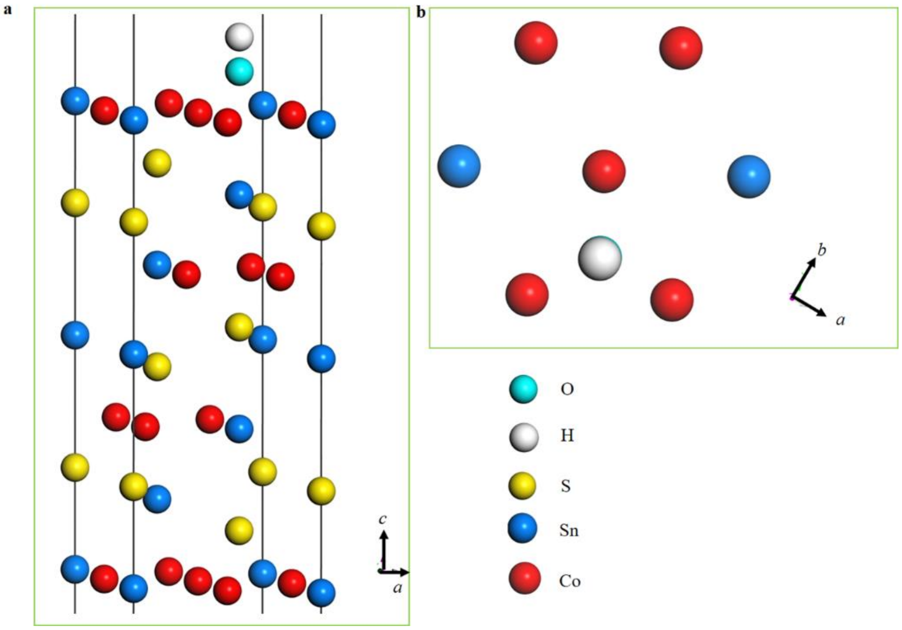

Fig. S16. The adsorption position of OH group on the crystal surface (surface plane perpendicular to the *c* axis). **a.** side view, and **b,** top view. It can be seen clearly that the OH group is adsorbed on the Co Kagome lattice.

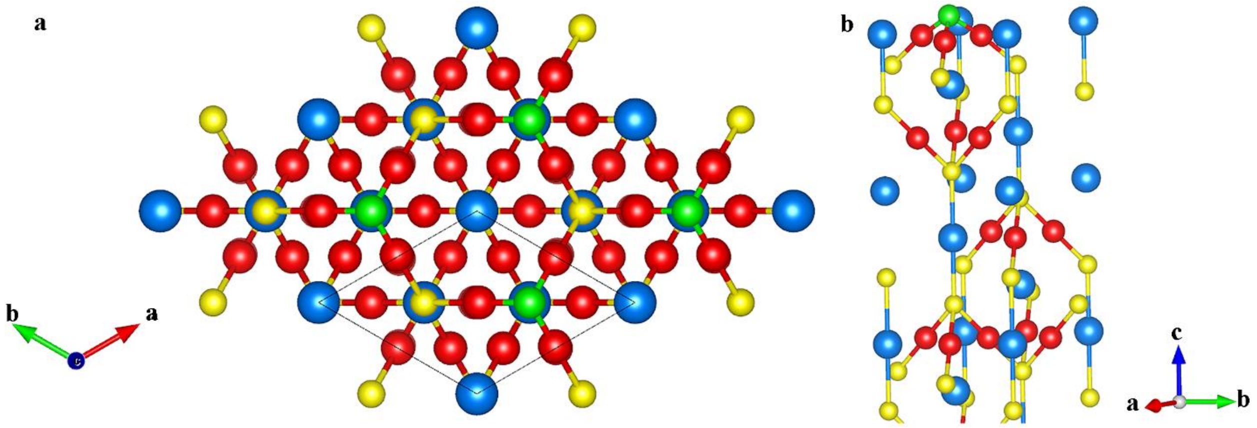

Fig. S17. The adsorption position of H atom on the $Co_3Sn_2S_2$ single-crystal surface. a. top view, and b, side view. It can be seen clearly that the hydrogen atom is adsorbed on the Co Kagome lattice. Blue balls are Sn atoms, yellow balls are S atoms, red balls are Co atoms, and green balls are hydrogen atoms. The Co Kagome lattice terminated (001) surface shows that it has a strong bonding with the hydrogen atoms. They are just located at the top of three Co atoms to form a tetrahedron. A Bader charge analysis carried out on H / $Co_3Sn_2S_2$ system revels that electron is transferred from Co atom to the H (up to 1.62 on H).
